# Effects of Hexane Root Extract of* Ferula hermonis* Boiss. on Human Breast and Colon Cancer Cells: An* In Vitro* and* In Vivo* Study

**DOI:** 10.1155/2019/3079895

**Published:** 2019-07-15

**Authors:** Nael Abutaha, Fahd A. Nasr, Mohammed Al-zharani, Ali S. Alqahtani, Omar M. Noman, Mohammed Mubarak, Semlali Abdelhabib, Muhammad A. Wadaan

**Affiliations:** ^1^Bioproducts Research Chair, Department of Zoology, College of Science, King Saud University, Riyadh, Saudi Arabia; ^2^Medicinal Aromatic and Poisonous Plants Research Center, College of Pharmacy, King Saud University, Riyadh 11451, Saudi Arabia; ^3^Al Imam Mohammad Ibn Saud Islamic University (IMSIU), College of Science, Biology Department, Riyadh, Saudi Arabia; ^4^Electron Microscope Unit, King Saud University Medical City, Riyadh, Saudi Arabia; ^5^Groupe de Recherche en Écologie Buccale, Faculté de Médecine Dentaire, Université Laval, Québec, Québec, Canada

## Abstract

Breast and colon cancers are leading causes of cancer-related deaths globally. Plants are a potential source of natural products that may be used for the treatment of cancer.* Ferula hermonis* (FH) is reported to have diverse therapeutic effects. However, there are few reports on the* in vitro* anticancer potential of FH extract. Our results showed that the* Ferula hermonis* root hexane extract (FHRH) can induce dose-dependent cytotoxic effects in breast and colon cancer cells with MTT IC_50_ values of 18.2 and 25 *μ*g/ml, respectively. The FHRH extract induced apoptosis in both breast and colon cancer cells; this was confirmed by light and nuclear staining, q-PCR, and caspase 3/7 activation. This study also demonstrated the antitumor activity of FHRH in 9,10-dimethylbenz[*α*]anthracene DMBA-induced rodent mammary tumor model. The GC/MS analysis revealed the presence of 3,5-Dimethylbenzenemethanol, Alpha-Bisabolol, Alpha-pinene, Beta-pinene, and Baccatin III that have various pharmacological potentials. Overall, the present study suggests that FHRH extract possesses anticancer potential which is mediated through apoptotic effects in MDA-MB-231 and LoVo cells. The present study also considered a basis for further investigations into the potential use of FHRH extract as an anticancer therapy for breast and colon cancers.

## 1. Introduction

Breast and colorectal cancers have been and still are the most prevalent cancers worldwide, with an estimated 2.4 and 1.7 million incident cases, respectively, in 2015 [1]. Both breast and colon cancers rank among the top cancers in the Saudi population, according to the Saudi Cancer Registry reports [2].

Due to this rising threat and the drawbacks of different treatment methods including high cost, poor availability, toxic side effects, limited efficacy, and resistance to anticancer agents, the search for novel and effective chemotherapeutic agents with high efficiency and low toxicity is of key importance. About 40% of marketed drugs are isolated from natural sources [3]. Cancer and infections are the two major therapeutic areas of drug discovery research programs, which are based on natural products [4]. Among all the research programs in different therapeutic areas in 2008, approximately 108 research programs were plant-derived natural products for drug discovery. Among these projects, about 2 were in preregistration phase, 5 were in phase III, 41 were in phase II, 14 were in phase I, and 46 were in preclinical stage of drug discovery [4]. There are many examples of anticancer drugs developed from plant sources, such as pomiferin isolated from* Maclura pomifera*, sulforaphane isolated from broccoli, thymoquinone isolated from* Nigella sativa*, and paclitaxel isolated from the bark of* Taxus brevifolia* [5].

Of note,* F. hermonis* (FH) is a small shrub that belongs to the Apiaceae family. The Apiaceae is a large family of aromatic flowering plants growing in temperate areas. There are about 300 genera and 3000 species of this family worldwide. This family comprises commonly used vegetables and medicinal herbs such as anise, coriander, caraway, fennel, carrot, celery, and parsley. Pharmacological reports have revealed that* F. hermonis *has several bioactivities which are useful to treat erectile dysfunction, menopausal disturbances, osteoporosis, skin infections, gastric disorders, fever, dysentery, neurological disorders [6], and diabetes [7]. The plant is also reported to have antimicrobial, anti-inflammatory [8, 9], and anticancer activities [8, 10]. An important hallmark of cancer cells is the evasion of apoptosis [11]. However, most of the existing chemotherapies work by inducing apoptosis as well as by producing direct toxicity. Since many plant extracts exert their effects through apoptosis [12], evaluating the ability of plant extracts to induce apoptosis is crucial. Therefore, in this study, we focused on the apoptotic mechanism evoked by the FH extract to combat diseases, which might help understand the toxicity-inducing mechanisms that mediate the action of the FH extract. However, the* in vivo* effect of FH extract on rats is not studied so far. To this end, the present investigation was designed to utilize a chemically induced breast carcinoma in rats by using 9,10-dimethylbenz[*α*]anthracene (DMBA), a tumor initiator that is being used for many years in cancer research to induce tumor development [13, 14] in order to characterize the potential effect of FH extract as a chemotherapeutic agent for treating breast cancer. To the best of our knowledge, only a few reports have been published on the cytotoxic activity of FH extracts. The present study shows for the first time the effect of the FH root extract on the DMBA-induced rat mammary carcinoma model, including the induction of cellular apoptosis, and inhibition of proliferation and migration of MDA-MB-231 and LoVo cells.

## 2. Materials and Method

### 2.1. Collection and Preparation of Plant Extracts

*Ferula hermonis* was procured from the markets of Riyadh, Saudi Arabia. The plant was authenticated (voucher No. KSU/2101) and identified by Dr. Jacob Thomas, Taxonomist, Department of Botany and Microbiology at King Saud University. The plant root was dried and ground using an electric mill. Fifty grams of root plant powder was weighed and then placed in a thimble in the Soxhlet extraction apparatus. The extraction process was performed for 24 h using hexane solvent (450 ml). The extract was collected, centrifuged at 5000 rpm for 10 min, and concentrated using a rotary evaporator (Heidolph, Germany) at 45°C. The concentrated extracts were stored in glass vials at -84°C until use. The dry weight of the hexane extract was dissolved in cell culture grade DMSO (Sigma) at 100 mg/ml stock.

### 2.2. In Vitro Study

#### 2.2.1. Cell Lines and MTT Proliferation Assay

Human breast adenocarcinoma (MDA-MB-231) and colorectal cancer (LoVo) cells were obtained from the Leibniz Institute DSMZ-German Collection of Microorganisms and Cell Cultures (Braunschweig, Germany). Cells were grown using Dulbecco Modified Eagle Medium with 10% Foetal Bovine Serum (Gibco, USA) and incubated in a humidified incubator at 37°C with 5% CO_2_. For experiments with FH extracts, the cells (5 × 10^4^ cells/well) were seeded in 24-well plate (Corning, USA) for 24h. The cells were then treated with different concentrations (0-100 *μ*g/mL) of FH extract for 48h. Cell viability was assessed by a standard colorimetric 3-(4,5-dimethylthiazolyl-2)-2, 5-diphenyltetrazolium bromide (MTT) assay as previously described [15]. To assess the percentage of cell viability in response to the FH extracts, the following formula was used: % cell viability= mean absorbance in test well/mean absorbance in control well x 100. IC50 values were determined for each cell line and used in the following experiments.

#### 2.2.2. Lactate Dehydrogenase (LDH) Assay

The LDH release assay was performed using an* in vitro* toxicology assay kit (Sigma, USA). Briefly, LoVo and MDA-MB-231 cells (about 5 × 10^4^ cells/well) were seeded in 24-well plates (Corning, USA). Upon 24 h of incubation, the culture medium was replaced with fresh medium containing 18.2 and 25 *μ*g/mL (MTT IC_50_) of the extract against MDA-MB-231 and LoVo cells. After 48 h of treatment, the culture medium was collected and centrifuged at 2500 rpm for 4 min. Aliquots containing 100 *μ*l of the solution from each reaction media were incubated with 100 *μ*l of the reactant lactate dehydrogenase assay mixture. After 30 min of incubation at 25°C in the dark, the LDH release was measured at 490 nm using the ELISA plate reader (Biochrom, England). Cytotoxicity of the extract was determined by comparing LDH release with the negative control, and the graph was plotted using the optical density value.

#### 2.2.3. Fluorescence Microscopic Studies

The effect of FHRH extract on the nuclear morphological changes of LoVo and MDA-MB-231 cells was studied; 5 × 10^4^ cells/well were seeded into a 24-well plate. After 24 h incubation, the cells were treated for 48h with the extract at the concentrations of 18.2 and 25 *μ*g/mL against MDA-MB-231 and LoVo cells, respectively, and then stained with Hoechst 33258 (Sigma) as previously described [15]. Images of the stained cells were captured using a fluorescent microscope equipped with a digital camera (Zeiss, Oberkochen, Germany).

#### 2.2.4. Quantitative Real-Time PCR (q-PCR)

q-PCR was carried out to determine the level of changes in some selected genes following treatment with the FHRH extract. q-PCR analysis was performed as previously described [16, 17]. Briefly, MDA-MB-231 cells were seeded on 6-well plate (5 × 10^4^ cells/well) and incubated for 24h. Thereafter, cells were treated with FHRH (IC_50_=18.2*μ*g/ml) and DMSO vehicle control. After 48h, total RNA was extracted from treated cells using Trizol ™ reagent. The cDNA was synthesized from 1 *μ*g of RNA using Promega kit (Reverse Transcription System (100), Cat. No. A3500, USA). q-PCR was carried out on an Applied Biosystems 7500 Fast Real-Time PCR System (Life Technologies, CA, USA), using specific primer for each gene. GAPDH was used as the internal reference. The mRNA expression levels were calculated by fold change using the following equation: fold change= 2^−ΔCT^ and ΔC_T_= (C_T_ gene of interest - C_T_ internal control).

#### 2.2.5. Caspase-3/7 Detection

Caspase-3/7 activity was detected using the Cell Event Caspase-3/7 Green Detection Reagent (Life Technologies, Waltham, MA, USA). The working principle of the kit relies on the cleavage of the four-amino acid substrate DEVD conjugated to a fluorophore. When caspase-3/7 is activated in apoptotic cells, the DEVD peptide is cleaved, allowing the dye to bind to DNA, resulting in a fluorogenic response that can be detected at ~ 502/530 nm. For this purpose, the MDA-MB-231 and LoVo cells (5× 10^4^ cells/well) were seeded in 24-well plates and after 24 h the cells were treated with 18.2 and 25 *μ*g/ml, respectively, of the FHRH extract for 48 h at 37°C. The cells were washed with PBS, and a fresh medium was added (0.5 ml). The plates were incubated with 2 *μ*M of the caspase-3/7 detection reagent for 30 min at 37°C in the dark. Finally, the cells were photographed at 200X magnification under a fluorescent microscope equipped with a digital camera (Zeiss, Germany).

#### 2.2.6. Scratch Wound Healing Assay

Cell migration was measured using an* in vitro* wound healing assay. Briefly, MDA-MB-231 cells (5 × 10^4^ cells/well) were seeded into a 12-well plate and incubated at 37°C and 5% CO_2_ in an incubator, and the cells were allowed to grow to 80% confluence. Then, a scratch was made on the culture plate with a sterile 0.1 ml pipette tip to create a cell-free area. The cells were treated with the FHRH extract at the concentration of 9 *μ*g/ml and images were captured after at 0, 24, and 48 h. An inverted microscope Leica MC-170 HD camera (Leica, Germany) was used to capture images at a magnification of 200X. The time lapse images were analyzed using ImageJ. Each experiment was performed in triplicate. The relative migration ratio was calculated according to following [18]:

(1) Relative  Migration  Ratio=Distance  at  0  h−Distance  at  48hDistance  at  0h.

### 2.3. In Vivo Study

Fifteen healthy virgin female Wistar albino rats (*Rattus norvegicus*), aged 5–8 weeks and weighing 115 ± 15 g, were obtained from the Animal House at the College of Pharmacy at King Saud University, Riyadh. The animals were housed at a room temperature of 25 ± 2°C and a relative humidity of 45 ± 5 % under 12 h light/12 h dark cycles. The rats were separated into three groups, with five rats in each group. Mammary tumors were induced in groups I and II with a single subcutaneous injection of the previously described potential carcinogen, dimethylbenzanthracene (DMBA), in the right pectoral area [19]. After tumor palpation (8 weeks postinjection), group I animals remained untreated, while group II animals were injected with FHRH extract dissolved in sunflower oil at a dose of 100 mg/kg into the tumor itself. A single dose was administered every 48 h, for 10 weeks, according to the protocol described by Ziad et al. [20, 21], with slight modifications. The group III control animals were injected with the vehicle of the extract (sunflower oil). Mammary gland tissues were collected after euthanization by ketamine and xylazine overdose from all sacrificed rats. Tumor size was measured using a calliper and tumor volume was assessed using V = 0.5 × (a(b)^2^) formula, where “a” and “b” are the major and minor diameters of tumors, respectively [22]. Tissues were excised and evaluated for histological studies. The tissues from the three groups were fixed in formalin, processed, and embedded in paraffin wax. Sections of 5-*μ*m thickness were stained with haematoxylin and eosin (HE). Tissue sections were examined under a light microscope (Nikon Eclipse 80i, Tokyo, Japan) at 100X, 200X, and 400X magnifications.

### 2.4. Gas Chromatography-Mass Spectroscopy (GC-MS)

The root extract of* Ferula hermonis* was analyzed for its phytochemical constituents by GC-MS (TurboMass, PerkinElmer, Inc., Waltham, MA, USA). Elite-5MS column was used (30 m long, 0.25 *μ*m in thickness, and 0.25 *μ*m in internal diameter). The oven temperature program started from as 40°C (2 min) and was then raised to 200°C at 5°C min-1 rate (2 min). From 200°C, the temperature was raised to 5°C min-1 to 300°C (2 min). Mobile phase (Helium gas, 99.999 %) was used at 1.0 ml min-1. The injection volume was 1 *μ*l. The phytocompounds were identified using the database of the WILEY 237 Spectral libraries and National Institute of Standard and Technology (NIST, Maryland, USA).

### 2.5. Statistical Analysis

The* in vitro* results are expressed as means ± SD of 3 independent experiments with 2 or 3 replicates per experiment. The data sets were compared by the Student's t-test. Differences were accepted as being statistically significant at p values < 0.05.

## 3. Results

### 3.1. In Vitro Study

#### 3.1.1. MTT Proliferation Assay

**Figure 1:**
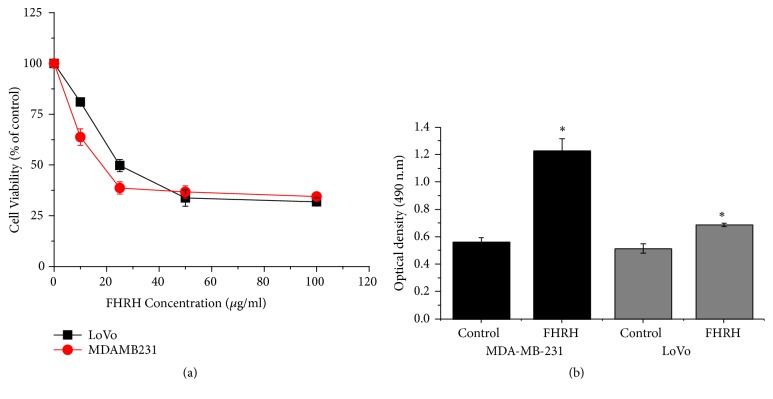
The effect of FHRH extracts in MDA-MB-231 and LoVo cells using MTT and LDH assays. (a) The absorbance of the MTT formazan was determined at 540 nm in an ELISA reader. (b) MDA-MB-231 (18.2 *μ*g/ml) and LoVo (25 *μ*g/ml) cells were treated with the FHRH extract for 48 h. LDH activity was determined at 490 nm in an ELISA reader. Data represent the mean ±SD (*∗*P< 0.05, *∗∗*P< 0.01, and *∗∗∗*P< 0.001 were considered significant compared to control) of three independent experiments carried out in triplicate. FH:* Ferula hermonis*, R: root, H: hexane.

The FHRH extract showed clear cytotoxicity on the MDA-MB-231 and LoVo cells by inhibiting viability in a dose-dependent manner, Figure 1(a). However, the LoVo colon cancer cell line was less sensitive compared to the breast cancer cell line, MDA-MB-231, with IC_50_ values of 25 and 18.2 *μ*g/ml, respectively.

#### 3.1.2. LDH Assay

The FHRH extract was cytotoxic and induced significant cell death in the MDA-MB-231 and LoVo cells, Figure 1(b). The cytotoxic effect of the FHRH extract was measured based on the intracellular LDH enzyme released into the culture medium upon plasma membrane damage. A significant release (p ≤ 0.05) of the LDH enzyme was observed after 48 h of treatment of the breast cancer MDA-MB-231 and colon cancer LoVo cells with the FHRH extract at concentrations of 18.2 and 25 *μ*g/ml, respectively.

#### 3.1.3. FHRH Alters the Nuclear Morphology of MDA-MB-231 and LoVo Cells

**Figure 2:**
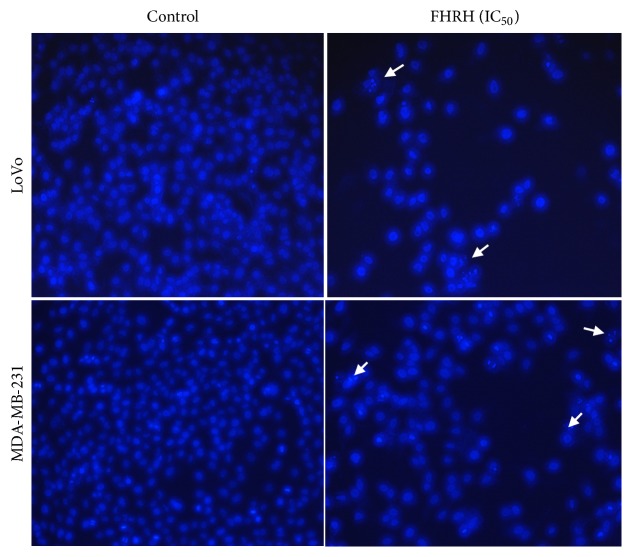
Nuclear morphology following FHRH treatment. Cells were stained using Hoechst 33258 to observe nuclear changes and photographed with fluorescence microscopy. Arrows indicate the cells with DNA fragmentation (magnification: 200 x).

Hoechst 33258 staining of the MDA-MB-231 and LoVo cells showed a light blue, homogeneous, and regular intact nucleus with minimal cellular debris in the DMSO-control group. The nuclear morphology of FHRH-treated cells was distinguished by the presence of damaged DNA with a brighter blue nuclear staining. The images showed apoptotic features such as nuclear DNA fragmentation and apoptotic body formation. The number of cells in the treated groups was also lower than that in the untreated group, Figure 2.

#### 3.1.4. Real-Time Polymerase Chain Reaction

**Figure 3:**
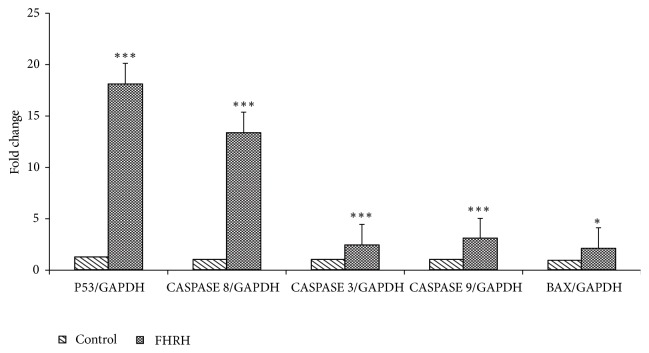
Effect of the FHRH extract on the mRNA expression of apoptosis-associated genes. Fold change of p53, Bax, and caspases 3, 8, 9 genes in MDA-MB-231 cancer cells treated with IC_50_ concentrations (18.2 *μ*g/ml) for 48 h. *∗*P<0.05, ^*∗∗*^P<0.005, and *∗∗∗*P<0.0005 compared to the control cells (DMSO vehicle). Data are presented as the mean ± standard deviation in the three independent experiments carried out in triplicate.

The gene expression analysis showed upregulation of p53, Bax, and caspases 8, 9, and 3, in the breast cancer cell line (MDA -MB-231) treated with the FHRH extract, where the fold changes were 18.08 ± 2, 2.07 ± 0.07, 13.4 ± 0.4, 3.07 ± 0.3, and 2.43 ± 0.2, respectively, compared to the untreated control group where the mean changes were 1.30 ± 1.2, 0.9 ± 0.11, 2 ± 0.7, 1.02 ± 0.3, and 1.02 ± 0.3, respectively. These increases were statistically significant (p ≤ 0.05, p < 0.005), Figure 3.

#### 3.1.5. Caspase-3/7 Detection

**Figure 4:**
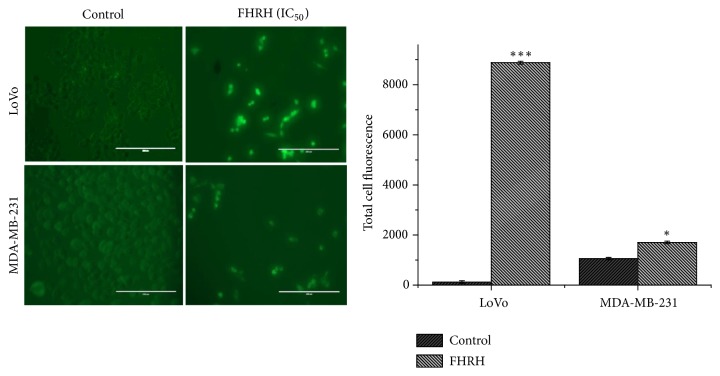
Detection of caspase 3 and 7 activity after MDA-MB-231 (18.2 *μ*g/ml) and LoVo (25 *μ*g/ml) cells treatment with FHRH extract for 48h. Caspase-3/7 activities were assayed by the CellEvent™ caspase-3′7 green detection reagent. FH:* Ferula hermonis*, R: root, H: hexane. (*∗*P< 0.05, *∗∗*P< 0.01, and *∗∗∗*P< 0.001 compared with the control (untreated cells)). Data are presented as the mean ± standard deviation of three independent experiments carried out in duplicate.

As shown in Figure 4, when cells were examined with the caspase-3/7 detection reagent for detecting apoptosis, bright fluorescent nuclei were observed in the MDA-MB-231 and LoVo cells treated with IC_50_ of the FHRH extract. In contrast, the untreated cells of the control group exhibited minimal fluorescence signal, since caspase-3/7 was not activated.

#### 3.1.6. Scratch Wound Healing Assay

**Figure 5:**
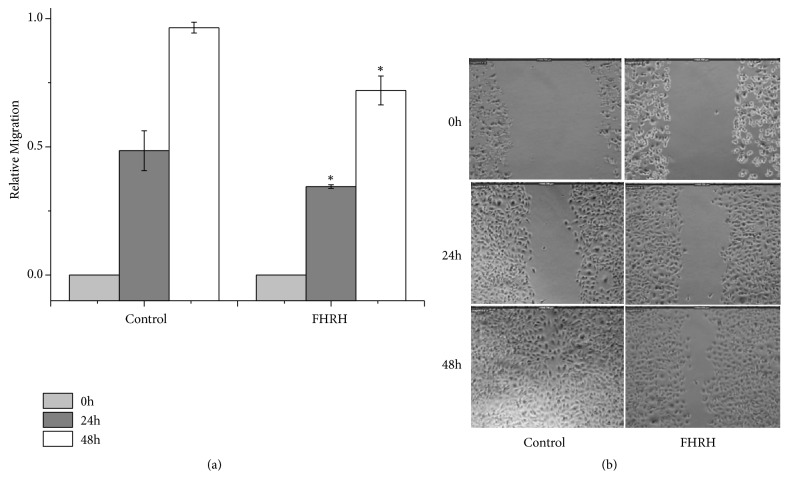
The effect of the FHRH extract on MDA-MB-231 cell migration. Cells were scratched with a pipette tip and then incubated with 9 *μ*g/ml of the FHRH extract for 48 h. Migrating cells were photographed under a phase-contrast microscope. FH: Ferula hermonis, R: root, H: hexane. *∗*P<0.05 compared with the control (untreated cells). Data are presented as the mean ± standard deviation of three independent experiments carried out in duplicate.

As shown in Figure 5, the wounding-healing assay revealed that the FHRH extract significantly inhibited the migration of MDA-MB-231 cells in a time-dependent manner (p ≤ 0.05).

### 3.2. In Vivo Study

#### 3.2.1. Histological Changes in Rats Treated with FHRH Extract after Tumor Induction

There was no significant decrease in tumor size after treatment with FHRH extract compared to control tumor bearing mice. An average tumor volume of 659.1 mm^3^ after 10 weeks was measured in untreated rat, while upon FHRH extract administration, the average tumor volume was 636.6 mm^3^ (Figure 7).

**Figure 6:**
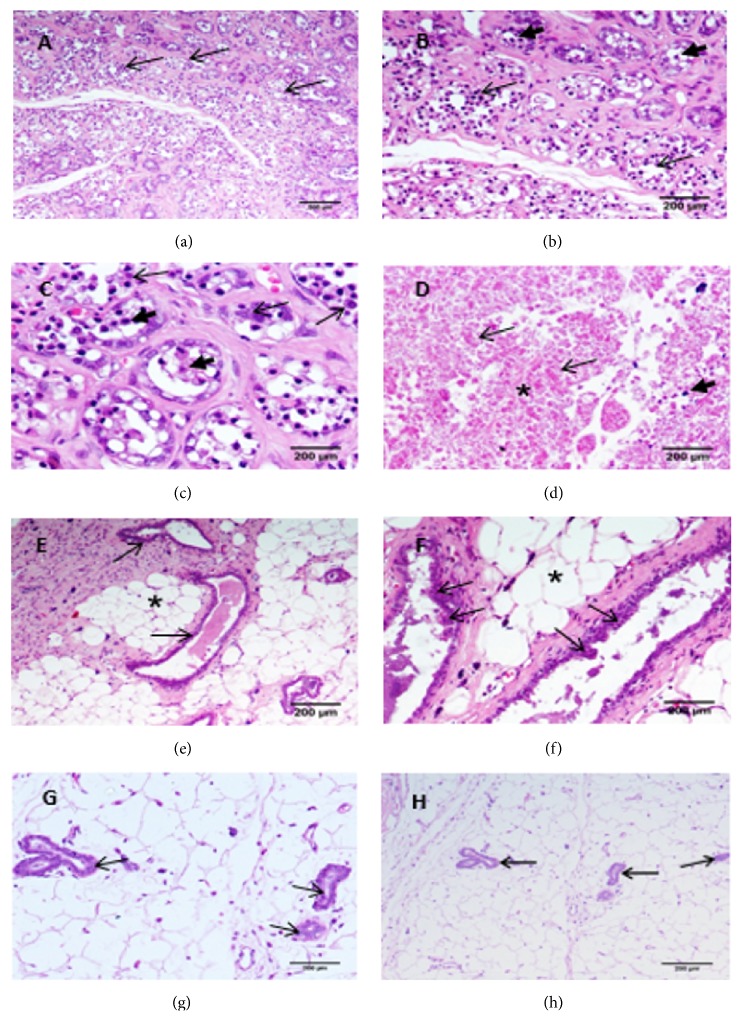
Light micrographs of tumor growths induced by DMBA (16 weeks postinjection) in mammary glandular tissue of female rats in comparison to rats injected with DMBA and then treated for 10 weeks with FHRH extract. (a) Proliferating neoplastic cells in the epithelial lining of mammary glandular ducts (small arrows). Neoplastic cells are also seen infiltrating the interstitial tissue. DMBA-injected animal. HE: 100X. (b) Neoplastic cell growth in the epithelial lining of mammary glandular ducts and presence of carcinomatous cells in the ductal lumina (arrowhead). DMBA-injected animal. HE: 200X. (c) Neoplastic cells in the ductal epithelial lining (arrows) and also in the ductal lumina (arrowheads). Note the nuclear pleomorphism of neoplastic cells. DMBA-injected animal. HE: 400X. (d) Area of necrosis (*∗*) in the mammary glandular tissue. Note the cytoplasmic and nuclear cell debris (arrows). DMBA-injected animal. HE: 100X. (e) Restriction of the proliferating neoplastic cells to a relatively limited number of mammary glandular ducts (arrows). Note absence of distinct neoplastic cell infiltration and also absence of remarkable areas of necrosis in interstitial tissue (*∗*). FHRH extract-treated animal. HE: 400X. (f) Proliferating neoplastic cells constituting only a few layers in the epithelial lining of mammary glandular ducts (arrows). No distinct areas of necrosis in the interstitial tissue (*∗*). (g-h) Normal structure of rat mammary gland tissue. Arrow indicates the ducts and epithelial lining cells. The adipose tissue (*∗*) surrounded the ducts (HE: 200x and 100x, respectively).

**Figure 7:**
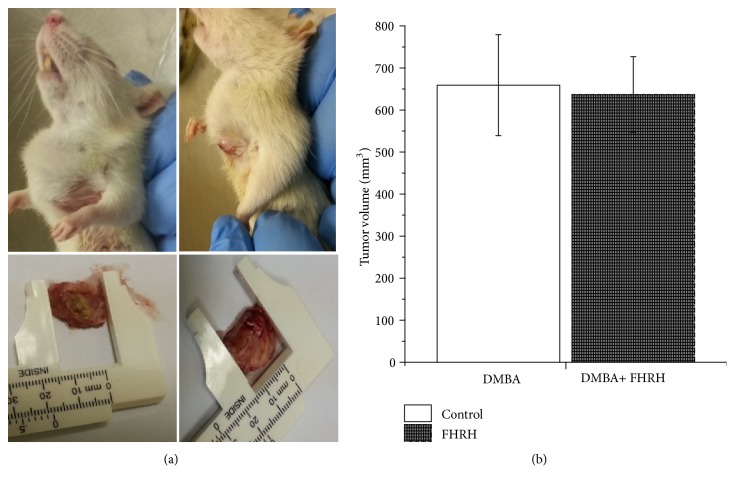
*Images represent the breast cancer tissue of in vivo experiment in rats*. Solid tumor was induced in Wistar albino rats (*Rattus norvegicus*) with a single subcutaneous injection by injecting 9,10-dimethylbenz[*α*]anthracene (DMBA). After 8 weeks postinjection, group I remained untreated, while group II were injected with FHRH extract at a dose of 100 mg/kg into the tumor itself. (a) Tumor size in DMBA group and after treatment with FHRH extract. (b) Tumor volume was calculated as described in materials and methods.

Light microscopy of the examined mammary gland tissues of the female white rats injected with DMBA revealed the development of carcinoma cells within the epithelial lining of the glandular ducts (Figure 6(a)). The epithelial lining affected by the neoplastic growth comprised several layers of the carcinomatous cells. The carcinoma cells were also discerned within the ductal lumen, causing varying degrees of luminal obliteration (Figure 6(b)). Some of these ductal lumina cells could not be easily identified due to complete obliteration by the proliferating carcinomatous cells. The carcinoma cells were characterized by nuclei of varied shapes and sizes (nuclear pleomorphism) with the existence of prominent nucleoli (Figure 6(c)).

Light microscopic examination also revealed the infiltration of interstitial tissues by large numbers of neoplastic cells. As a whole, the glandular alveoli of the mammary tissues in the DMBA-injected female rats showed remarkable disruption of the normal histological pattern due to massive growth and infiltration by the neoplastic cells. Large areas of necrosis (Figure 6(d)) were also observed along the neoplastic growth sites within the mammary glandular tissue. Areas of necrosis were identified by the presence of nuclear and cytoplasmic debris.

Histological examination of the mammary gland tissues of the female rats treated with FHRH after DMBA injection showed a marked reduction in the tissue areas affected by the neoplastic growth. This was evidenced by the absence of ductular structures obliterated by carcinomatous cells. The proliferating neoplastic cells were restricted only to a few layers, lining the ductal walls (Figure 6(e)). There was no noticeable infiltration of neoplastic cells within the interstitial tissues, and also no comparable areas of necrosis were noticed within the mammary glandular tissue (Figure 6(f)). On the whole, the main histological architecture of the mammary tissues of the female rats treated with the FHRH extract following DMBA injection was preserved, compared to the histological architecture of rats injected with DMBA alone.

### 3.3. GC-MC Analysis

**Figure 8:**
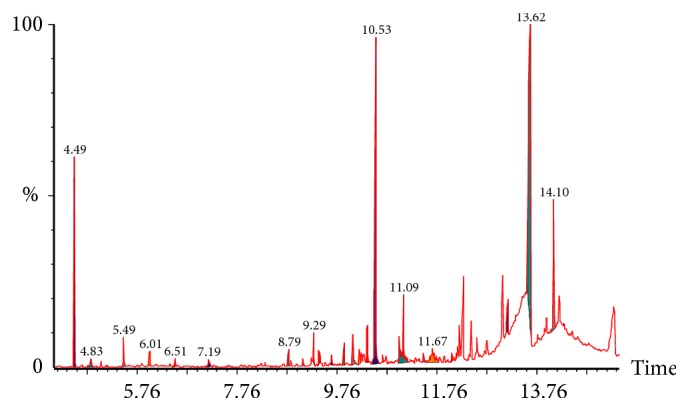
GC-MS analysis of the hexane extract of* F. hermonis*.

The phytocompounds present in FHRH extract with their elution time, molecular formula and the amount of these phytocompounds are shown in Table 1. The main compounds detected in the extract were 3,5-Dimethylbenzenemethanol (36.15%), Alpha-pinene (22.62%), Alpha-Bisabolol (15.92), and Baccatin III (6.57%). The GC chromatograms of FHRH extract are presented in Figure 8.

## 4. Discussion

Currently, finding the balance in cell proliferation appears to be the key factor for maintaining homeostasis by inhibiting unrestricted cell proliferation. This could take place by inducing apoptosis and arresting the cell cycle in cancer cells. There are an increasing number of studies focusing on plant extracts and their effect on the metastasis of different kinds of cancers cells. The uniqueness of this study lies in the fact that the* in vivo* anticancer properties of* F. hermonis* have never been reported before.

In this study, we also evaluated the anticancer properties of* F. hermonis* root extract against MDA-MB-231 (breast) and LoVo (colorectal) cancer cell lines. MTT and LDH assays are extensively used in* in vitro* toxicological methods to study the cytotoxic and negative impacts on cell viability. In order to increase the credibility of the results and to avoid misestimating the toxicity of the test extract, other tests need to be performed to assess cell viability in* in vitro* studies. The MTT and the LDH release assays were therefore performed in this study (Figures 1(a) and 1(b)). In this study, the FHRH exhibited anticancer activities by reducing the viability and proliferation of breast and colon cancer cell lines in a dose-dependent manner. LDH is a perfect marker for cytotoxicity studies because its release is an indication of irreversible cell death attributed to cell membrane damage [23]. The current investigation revealed that LDH leakage increased significantly (p ≤ 0.05) when breast and colon cancer cells were treated with the FHRH extract, as compared to the DMSO-control. Hence, LDH leakage might have been due to the cytotoxic nature of the plant extract, which therefore confirms its antitumor activity [24–27].

The National Cancer Institute (NCI) reported that when the IC_50_ of crude extracts against cancer cells is less than 30 *μ*g/ml, it is considered a promising extract for further research [28]. In the current study, it was found that the IC_50_ of the FHRH extract falls within this defined scope of NCI, therefore making the FHRH a promising extract for cancer chemotherapy. Agents that induce apoptosis can be perfect candidates for cancer chemotherapy. Various plant-derived products have been shown to induce apoptosis in many types of cancer cells [29, 30]. For over a century, morphological features played the leading role in describing cell death [31]. Apoptosis is characterized by a series of typical morphological features, such as cell shrinkage, formation of apoptotic bodies, blebbing, chromatin condensation, and nuclear fragmentations, which were observed in the current study using light and fluorescent microscopy.

In the present study, the MDA-MB-231 cells treated with root extract demonstrated the* in vitro* anticancer activity through the apoptotic pathway. However, the activation of the caspases, including caspases 3, 8, and 9, is implicated in both the intrinsic and extrinsic pathways of apoptosis. Recently, many studies have shown that the p53-inducible proapoptotic genes trigger apoptosis through both extrinsic and intrinsic apoptotic pathways [32, 33]. In addition, the induction of apoptosis was confirmed by fluorescent techniques using the caspase-3/7 detection reagent. Treatment with the FHRH extract resulted in bright fluorescent nuclei (Figure 4), a hallmark of cells undergoing apoptosis [34].

Metastasis is one of the most crucial threats to a successful cancer treatment. Therefore, compounds that prevent the migration of cancer cells are important for disease treatment and prognosis. The use of the FHRH extract as an anticancer agent is supported by the wound healing effect the extract exhibited against MDA-MB-231 cells, the highly invasive and aggressive cell line. To the best of our knowledge, there are no studies that report the inhibitory activity on cell migration of extracts from* F. hermonis.*

Daucane derivatives are some of the common constituents of various species of the genus* Ferula* [10, 35, 36]. Interestingly, these compounds are reported to cause inhibition of the human breast cancer cell line (MCF-7), bladder cancer cell line (TCC), pancreatic cancer cell line (MIA PaCa-2), and CCRF-CEM leukaemia cells [10, 37, 38]. Daucane derivatives are also known to activate caspases 3, 8, and 9, causing cell cycle arrest and DNA laddering [36, 39, 40]. Some of the daucane derivatives have been reported to be present in the* F. hermonis* root extract [41]. The cytotoxic activity might be attributed to these compounds; however, further experiments are needed to confirm our conclusion.

To the best of our knowledge, no previous studies have assessed the antiproliferative effect of* F. hermonis* root extract (FHRH) against MDA-MB-231 and LoVo cell lines, although one study reported the cytotoxic effect of* Ferula hermonis* root extract against the MCF-7 cell line [10]. Many studies report SD rats as a promising animal model which can produce encouraging results for chemoprevention [42, 43]. This study revealed that tumor induction by the polycyclic hydrocarbon 7,12-dimethylbenz(a)anthracene (DMBA) in female SD rats started in the 6^th^ week of administration. The features of this model are comparable to the human system, including similarities in histological progression and hormone dependence [44]. Human breast cancers usually originate in the ductal region, and here, the DMBA-induced mammary tumor model exhibited the same origin [45]. In the present study, the body weight of the rats did not show significant differences between normal and tumor-bearing rats, suggesting that our experimental model did not produce side effects that could induce weight loss (data not shown). Similarly, in studies reported by [46, 47], there were no significant changes in body weight between the control and experimental sets (data no shown). The FHRH extract decreased the size of the tumor, although no statistically significant differences were found. However, histopathological analysis of the extract-treated group (group II) showed that the mammary tissue was preserved in comparison to rats injected with DMBA alone.

The GC/MS analysis (Table 1 and Figure 8) revealed the presence of 3,5-Dimethylbenzenemethanol, Alpha-Bisabolol, Alpha-pinene, Beta-pinene, and Baccatin III that have various pharmacological potentials. Alpha-pinene is found in the essential oils of different plants and reported for its in vitro and in vivo anticancer, anti-inflammatory, and antioxidant activities [48, 49]. *α*‐Bisabolol is also found in a variety of plant extracts and is reported to induce apoptosis in different cancer cells and suppressed tumor growth in tumor‐bearing mice model [50]. Baccatin III is the specific precursor of Taxol that was isolated from* Taxus baccata* L. and known to induce apoptosis in different cancer cells [51–53].

In conclusion, the FHRH extract exhibited a cytotoxic effect on tumor cells, as evidenced by the inhibition of proliferation of the human breast and colon cancer cells. The extract also provoked a marked reduction in the mammary tissues affected by the neoplastic growth in DMBA-injected animals. As a whole, the main histological architecture of the breast tissues was preserved compared to rats injected with DMBA alone. This study shows the potential effect of the FHRH extract as an anticancer agent and paves the way for further research on FHRH extract in the field of anticancer therapy.

## 5. Conclusion

The present study suggests that FHRH extract possesses anticancer potential which is mediated through apoptotic effects in LoVo and MDA-MB-231 cells. FHRH extract is the most potent extract and further investigations on phytochemical are under way to isolate the active compound/s.

## Figures and Tables

**Table 1:** Compounds identified by GC-MS in the FHRH extract.

Compound Name	Chemical formula	MW (g/mol)	RT (min)	Area	Area %
*α*-Pinene	C_10_H_16_	136.238	4.49	8755879	22.620
*β*-Pinene	C_10_H_16_	136.238	4.83	236068	0.610
2,4-Dimethyl-2,3-heptadien-5-yne	C_9_H_12_	120.195	5.49	384428	0.99
(+)-cis-verbenol	C_10_H_16_O	152.237	6.01	168132	0.430
Bicyclo[3.1.1]Hept-3-en-2-one	C_7_H_8_O	108.14	6.51	125659	0.320
1,6,10-Dodecatriene	C_12_H_20_	164.292	8.79	579609	1.500
*α*-Curcumene	C_15_H_22_	202.341	9.08	96612	0.25
*β*-Bisabolene	C_15_H_24_	204.357	9.29	430819	1.11
(-)-*γ*-Cadinene	C_15_H_24_	204.357	9.39	287247	0.74
Spathulenol	C_15_H_24_O	220.356	9.90	353572	0.91
3A(1H)-Azulenol	C_15_H_26_O	222.372	10.07	287294	0.74
Bisabolol Oxide B	C_15_H_26_O_2_	238.371	10.36	542742	1.4
*α*-Bisabolol	C_15_H_26_O	222.372	10.53	6161554	15.92
3,5-Dimethylbenzyl alcohol	C_9_H_12_O	136.194	13.62	13990848	36.15
Baccatin III	C_31_H_38_O_11_	586.634	14.10	2542351	6.57

## Data Availability

The data used to support the findings of this study are included within the article.
